# Supplementary material for: Are carbon emissions trading and green financial instruments synergistic? -Comprehensive quantitative research based on content analysis
Source: PLoS One. 2024 Mar 7;19(3):e0298601. doi: 10.1371/journal.pone.0298601 (PMC10919601; doi:10.1371/journal.pone.0298601)
Supplement: S1 Data — (ZIP) [file pone.0298601.s001.zip › Data/地级市Z_F工作报告.zip!/║╙▒▒/╠╞╔╜╩╨/└┤╫╘╣1⁄2╓┌║┼í╛┬φ┐╦╩2╛▌═°í┐_2019.docx]

二〇一八年工作回顾

2018年是全面落实党的十九大精神、奋力谱写新时代唐山高质量发展新篇章的开局之年。一年来，我们以习近平新时代中国特色社会主义思想领航定向，在省委、省政府和市委坚强领导下，深入贯彻习近平总书记视察唐山重要指示，创新突破，实干担当，以稳中求进应对稳中有变，以破立并举提速转型升级，“三个努力建成”和“两个率先”迈出新步伐。

一年来，我们以不负嘱托的使命责任接续奋斗，开新局、谱新篇;以知难而进的魄力担当破旧立新，治污染、促转型;以不忘初心的为民情怀提升福祉，增民利、暖民心;以干则必成的决心韧劲一抓到底，重实效、创一流。

一是优质新增量调优新旧占比。按季度组织项目集中开工和观摩测评，217个转型支撑重点项目完成投资1466亿元，中冶瑞木新材料等一批重大项目建成投产。新增规模以上工业企业238家、规模以上服务业企业180家，占全省新增总量的17.6%和29.7%，分列全省第1位和第2位。工业技改投资、战略性新兴产业投资分别增长25%和26%，高新技术产业对规模以上工业增长贡献率超过23%。京唐城际、水曹铁路等重大基础设施项目提速建设，唐廊高速建成，京秦高速二期通车，5条“公转铁”专用线改造完成。唐曹铁路客货运通车运营，结束了曹妃甸没有通达旅客列车的历史。省级以上开发区主营业务收入突破1.5万亿元，税收占全市比重提高到85.5%，成为增比进位突破的发展高地。

二是创新竞进撬动产业迭代升级。主动化解环境容量过剩产能，全年压减炼钢产能500.25万吨、炼铁产能298万吨、焦炭产能185万吨、煤炭产能201万吨、平板玻璃产能300万重量箱。坚持把科技创新变成实实在在的产业活动，新增科技型中小企业1423家、总数达到6654家，新增高新技术企业232家、超过近5年增量总和。全市规模以上工业企业利润增长20%，战略性新兴产业增加值增长17.2%。成功举办首届中国(唐山)工业设计展、全国钢结构装配式建筑设计创新竞赛等活动，中车唐车公司荣获中国优秀工业设计奖金奖。全年培训各类企业管理人才8000多人次，特别是组织4批次83名企业家赴德国、美国、日本学习培训，实现了开阔眼界、启智创新、促成合作的多赢。成功创建国家农产品质量安全市，农业产业化经营率提高到65.6%。

三是开掘优势潜能加速港产城融合。唐山港货物吞吐量6.37亿吨、跃升全国沿海港口第3位，集装箱吞吐量295.8万标箱、增长16.9%。曹妃甸国家级石化产业基地总体规划获批。站西、东湖、国丰北区、城南等重点片区强势启动，中国铁路源头博物馆、金达纪念馆、汉斯·昆德故居、唐山饮食文化博物馆(唐山宴)、中国(唐山)皮影主题乐园建成运营，南湖灯光秀惊艳全国，唐山国际旅游岛获批全省首家省级旅游度假区，“唐山周末”成为城市新名片。成功举办唐山开埠140周年嘉年华、第二届中国工业旅游产业发展联合大会、第三届中拉沙足锦标赛、第三届中国国际房车旅游大会、中国国际商标品牌节、全国首届地震科普大会等80余项赛事展会，旅游、会展业收入分别增长26%和28%。新型城镇化和美丽乡村建设不断提速，全市常住人口城镇化率提高到63.1%。

四是破除堵点释放改革开放新活力。全面打造“四最”唐山品牌，市级行政审批事项由229项精简到90项，行政性事业收费项目由38项降为26项，推广实施联合审批、“多评合一”“打包审批”等制度，压缩项目审批时限20%以上。新增市场主体12.75万户、中小微企业2.36万户，分别增长27.3%和10.2%。创新推行“政府主导、市场运作、连片成方、综合开发”经营城市新模式，有效破解以往棚改因一二级土地市场不分导致的拆迁成本难量化、群众回迁难等问题。盘活工业用地遗留问题7168亩，47个项目打通融资与上市的瓶颈制约。成功获批全国第三批跨境电子商务综合试验区，网易友品总部落户运营。迁安市化解钢铁过剩产能、推动产业转型升级典型经验获国务院通报表扬。新设山西大同、甘肃嘉峪关等内陆港12个，新开通日本东京等2条外贸集装箱班轮航线，开行曹妃甸港区—蒙古乌兰巴托、京唐港区—比利时安特卫普国际班列。唐山机场旅客吞吐量57.4万人次、增长10.6%，货邮行吞吐量4372吨、增长12.8%。

五是治本攻坚消除污染顽疾。坚持顶层设计、标本兼治、系统推进、重点突破的统筹观念，强力推进污染退城、清水润城、城市增绿，以“清准高快狠”的务实举措狠抓工业企业治理、扬尘治理、散乱污出清等“六个专项行动”，推动“点上治标”转向“全面治本”。全年PM2.5平均浓度达到60微克/立方米、同比下降9.1%，达优天数增加11天，重度污染以上天数减少17天，优增劣减、蓝天“含金量”提高。坚持决心一次下足，措施一步到位，启动钢铁企业整合搬迁和280家陶瓷企业退城进园，强势开展无名场院、违规建筑清理整治，排查散乱污企业5923家、取缔拆除4498家。河道治理生态修复由迁西长河拓展到唐山全域，主城区和入海河流断面消除劣五类水。铁路疏港矿石1500万吨，是2017年的2.5倍。完成462公里“一环四线”通道绿化和92公里城市绿廊建设，森林覆盖率提高到37.9%。

六是成果共享增强群众获得感幸福感安全感。2565户、6663名建档立卡贫困人口高质量脱贫，城镇和农村居民人均可支配收入分别增长8%、9.1%，城镇登记失业率2.42%。4.1万套棚改安置住房全部开工，197个老旧小区、242条背街小巷完成改造，城市二环线全线贯通，13条断头路、8条拥堵路打通畅行。新建改扩建中小学108所、幼儿园71所，妇幼保健院新院区、南湖医院开诊运营，京唐乌拉圭国际足球学校、南湖足球公园和足球主题酒店正式投用。新改建农村公路435公里，40.2万人农村饮水安全工程改造提标。滦县撤县设立滦州市。村、居“两委”换届圆满完成。平安唐山建设强力推进，“治乱、治电、治污、治车、治赌”等攻坚行动成效显著，扫黑除恶专项斗争攻势凌厉，线索核查、打处总量全省领先，打击质量连续两年全省第一，有效排查化解安全生产、食品药品、金融、非法集资、房地产、信访稳定等领域的风险隐患，社会大局和谐稳定。电视连续剧《那座城这家人》生动再现了英雄的唐山人民自强不息、感人肺腑的奋斗乐章，社会各界感同身受、纷纷点赞。

七是从严治政服务高质量发展。坚决贯彻全面从严治党规定要求，增强“四个意识”，践行“两个维护”，扎实开展“不忘初心、牢记使命”主题教育，严肃党内政治生活，不折不扣抓好中央和省委巡视反馈意见整改落实。调整优化机构编制，市县两级机构改革基本完成。认真执行市人大及其常委会决议，自觉接受各方面监督，360件人大代表建议、557件政协委员提案全部办复。严格落实中央八项规定及其实施细则，坚决遏制“四风”反弹回潮，全市“三公”经费下降5.1%，创新、一流、务实、高效、担当成为政府系统干事创业的时代新风。国防动员、民族宗教、新闻出版广电、外事侨务、妇女儿童、人民防空、老区建设、气象地理、史志档案、老龄、残疾人等各项事业取得新成效。

2019年政府工作总体要求

今年是新中国成立70周年，也是唐山加快“三个努力建成”步伐、实现“两个率先”目标的关键之年。前不久召开的市委十届六次全会，明确提出今年是“高质量发展突破年”，全市上下要牢记使命、担当实干，创新突破、奋勇争先，朝着“三个努力建成”和“两个率先”目标加速前进。

今年政府工作的总体要求是：以习近平新时代中国特色社会主义思想为指导，围绕加快实现“三个努力建成”和“两个率先”目标，坚持稳中求进工作总基调，坚持新发展理念，坚持以供给侧结构性改革为主线，坚持深化市场化改革、扩大高水平开放，开展“高质量发展突破年”活动，深化“四项战略”，打好“五场硬仗”，全力推进生态唐山、环渤海地区新型工业化基地、“一港双城”、港口和海洋经济、重大项目建设和科技创新、乡村振兴、人才队伍、文化旅游产业融合发展、优化营商环境、教育和医疗康养高地建设“十个新突破”，为全面建成高质量小康社会打下决定性基础，加快建设开放创新包容、宜居宜业宜游的现代化国际滨海城市。

2019年目标任务和工作安排

今年经济社会发展的主要目标是：地区生产总值增长7.5%左右，一般公共预算收入增长7.5%，规模以上工业增加值增长6.5%以上，固定资产投资增长7%以上，社会消费品零售总额增长10%，服务业增加值增长10%;新增规模以上工业企业230家以上、规模以上服务业企业170家;城镇和农村居民人均可支配收入分别增长8%、8.5%;每万元生产总值能耗和化学需氧量、二氧化硫、氨氮、氮氧化物减排完成省达任务。上述目标的设定，兼顾了速度与质量的协调，统筹了当前与长远的衔接，考虑了需要与可能的平衡，经过努力是完全可以实现的。

落实市委“高质量发展突破年”部署要求，必须聚焦“高质量”和“突破”两个关键词，精准发力、务实推进。今年，重点抓好十方面工作：

1.强化有效投资和项目支撑，巩固稳中有进、持续向好的强劲态势

一是以抓实新开工亿元以上项目、新增规模以上企业“两新”导向调结构优动能。用新开工亿元以上项目衡量项目建设是不是抓实了，用新增规模以上工业企业和服务业企业衡量项目建设是不是抓成了。严格项目准入，突出亩均投入、亩均税收、能耗水平、集约用地等关键指标，以优化投资结构调整产业结构。二是以加快基础设施建设补短板稳投资。实施重大基础设施项目44个，完成投资584亿元。用好“蓝天基金”，16条“公转铁”专用线建成通车，打通工业经济“低碳走廊”。三是以提速开发区发展强支撑促转型。以高端高新和立市立县大项目为重点，深入开展产业链招商、小团组招商、以商招商和“凤还巢”工程。完成开发区人事薪酬制度改革，填满装实海港中东欧产业园、中国(乐亭)拉美产业园等中外合作园区，打造对外开放、项目招商的新引擎。四是以扶持优质企业提质量增活力。毫不动摇支持民营经济高质量发展，常态深入企业一线排忧解难，组织开展“批而未供”“供而未用”土地清理专项行动，高质量完成全国第四次经济普查任务，支持企业通过多层次资本市场降低融资成本。

2.聚焦环渤海地区新型工业化基地建设，加快打造“4+5+4”现代产业体系

一是以“四个一”行动加快传统产业迭代升级。在钢铁等传统企业深入开展“引进一个专家团队、建立一个研发机构、选投一个战略性新兴产业、实施一个高管培训计划”等“四个一”行动，通过引进高端人才、先进技术催生新产品新产业新业态。二是以集群发展加快新兴产业倍增突破。深入实施《唐山市战略性新兴产业发展三年行动计划(2018-2020年)》，滚动实施100个新兴产业项目，年度投资100亿元以上，高新技术产业增加值增长15%。三是以业态融合加快产业价值链向高端跃升。以服务增值带动产业升级，年内现代物流、现代金融、新兴信息服务和研发设计等四大生产性服务业增加值增长15%以上。

3.深入实施创新驱动发展战略，打造唐山“双创”升级版

一是提高产业创新能力。深入实施高新技术企业和科技型中小企业双倍增计划，促进优势科技资源和创新服务向企业集聚。支持各县(市、区)开展科技招商，借力京津布局一批特色鲜明的“微中心”和创新产业园，构建“研发在北京、转化在唐山”“京津孵化、唐山产业化”的协同创新格局。二是集聚创业创新人才。深入实施“凤凰英才”计划和科技英才“双百双千”工程。精准开展分行业、小团组企业家培训，全面激发民营经济发展内生动力。三是优化创业创新生态。鼓励企业加强与科研院所、产业联盟、创投基金合作，创办市场化专业化创新平台，实现产学研资融合、创业创新创富一体。加大政府产业引导基金、科技风险投资基金对合伙人的让利力度，支持科研人员技术入股、兼职取酬。对外引内生的高端人才、高新企业，实行领导对接包联、部门订制服务，营造舒心放心的创业创新环境，打造企业成长沃土、创业圆梦福地。

4.全力打造沿海经济增长极，做强高质量发展“蓝色引擎”

一是唐山港转型增效。加强岸线资源、码头泊位、航线口岸的统筹管理，实现规划、建设、运营、品牌“四统一”。构建“口岸+市场+贸易”增值链条，争列平行车进口试点，提速曹妃甸矿石混配等大宗商品交易中心建设，积极培育“唐山港价格指数”。二是临港产业提质升级。滨海地区实施亿元以上项目287个，年内完成投资670亿元以上。发挥滨海优势，培育引进休闲度假、健康养生、文化创意等产业项目。三是海洋经济提速扩量。深挖“海上富矿”，建设海洋科技开发示范和中试基地，深耕“海上粮仓”。四是沿海板块进位突破。举全市之力支持曹妃甸高质量发展，全面深化园区管理体制机制改革，京冀(曹妃甸)协同发展示范区引进超亿元京津合作项目100个以上。芦台开发区、汉沽管理区全力推进津冀协同发展示范区建设。支持海港开发区、乐亭县、滦南县、丰南区培育壮大特色海洋经济和临港产业，打造沿海经济带重要支撑。

5.优化“一港双城”战略布局，加快建设现代化国际滨海城市

一是以高水平规划提品位。对标雄安新区和天津滨海新区，加快推进城市总规、控规等7方面35项规划修编，构建“城市规划+城市设计+建设导则”的全链条规划体系。二是以重点片区建设提升主城区首位度。南湖片区实施智能微轨观光、国际异域风情街等重点项目，南湖CBD一期局部封顶，核心区改造提升工程基本完成，打造独具魅力的“城市会客厅”，站西片区打造彰显唐山新形象的“城市门户”，东湖片区打造生态修复、变废为宝的“城市后花园”。抓好丰南国丰北区、路南城南经济开发区、高新京唐智慧港和路北秦黄下片区建设，加速开平、丰南融入主城区。三是以破堵点强弱项撬动城市有机更新。强力推进“三改一通”工程，实施38个棚户区、248个老旧小区和189条背街小巷改造提升，打通42条断头路、破损路，渠化治理32个拥堵路口，二环线与唐丰快速路互连互通，打造外快内畅的交通体系。加快南湖国际会展中心南区建设，新体育中心“两馆一场”年内主体完工。完成新华道、建设路等7条主干道“一街一景”建设，市中心区新建24个街头游园、15个以上“口袋公园”。加快西郊、东北郊污水处理厂迁建，实施既有建筑节能改造367万平方米，各县(市、区)建筑垃圾消纳处理设施投入运营。新建唐山西综合客运枢纽，实现铁路、长途汽车及公交客运一站式换乘。四是以融合联动思维加快建设曹妃甸滨海新城。释放港产城教融合发展潜能，优化临港现代工业、高新技术、港口商务等功能区设置，沿海各县(区)与曹妃甸新城统筹规划、错位发展，共建靓丽城市、生态港湾。

6.提速文化旅游产业融合发展，厚植城市转型新支撑

一是推动全域旅游增内涵提效益。围绕创建省级全域旅游示范市，实施文旅竞争力提升计划，拓展畅游海岛湿地、观览皇陵长城胜迹等15条精品线路，抓好恒大童乐园、遵化恋乡小镇等27个重点项目。放大“唐山周末”品牌知名度影响力，办好以滦河文化为主题的第三届全市旅游产业发展大会，加快唐山国际旅游岛创建国家级旅游度假区，启动长城旅游公路升级改造、南部滨海风景大道规划建设。二是打造工业旅游城市经典和消费热点。坚持以重塑文化价值传承工业文脉，启动老交大文化园、老火车站周边改造等重点项目，建设培仁女中记忆馆，培仁里历史文化街区“五一”前主体运营。以产业思维、市场方式抓好中国(唐山)工业博物馆、中国唐山陶瓷博物馆、中国铁路源头博物馆、启新1889中国水泥博物馆、开滦国家矿山公园等文旅项目运营管理。支持南湖与开滦国家矿山公园联合创建5A级景区，办好第三届中国工业旅游产业发展联合大会。三是以“旅游+”“文化+”激活新业态新动能。全力办好国际钢铁冶金博览会、国际体博会等100项赛事展会活动。精心承办6月份第二届中荷友好省市大会，筹办2020年第七届中法地方政府合作高层论坛。高质量举办中国国际应急管理大会、第二届全国地震科普大会，务实推进唐山应急装备制造产业园建设。以唐山演艺集团搬迁南湖影视基地为契机，打造既叫好又叫座的唐山戏曲文化园。办好第四届中拉沙足锦标赛、中国国际友好城市青少年足球邀请赛，发挥京唐乌拉圭国际足球学校的牵引作用，激活释放南湖足球公园、足球广场和南湖足球主题酒店的独特优势，以打造“足球城市”彰显城市个性魅力。

7.坚持改革开放再出发，全面激发转型发展动力活力

以改革之手删繁就简，激发内生动力。以群众满意为标准，深层次推进“放管服”改革，设立政务服务“午间岗”“AB岗”，完善上门服务、预约服务、代办服务等制度措施，动态打造“四最”唐山品牌。以减放结合提速、简并联动提效，在全省率先实施工程建设项目审批全流程、全覆盖改革。健全完善市属国有企业业绩与薪酬联动的评价体，实现由管企业向管资本转变。唐重集团和冀东集团完成重组，深化农村供销社综合改革，加快农垦改革，柏各庄、芦台、汉沽农场组建区域性现代农业企业集团。以开放之手集聚要素，提速转型嬗变。以跨境电子商务综合试验区为引爆点，全力打造中国北方跨境电商总部基地。设立2个境外产品展示中心，建立5个公共海外仓，培育本土跨境电商重点企业10家，引进国内外跨境电商企业10家，跨境电子商务交易额突破100亿元。国际邮件互换局、国际快件监管中心尽快获批。调整优化唐山机场航线航班，加快航空口岸开放申报。实施外贸质量对标提升行动，发挥国际友城和外侨社团组织纽带作用，稳步推进国际并购和产能合作。

8.狠抓污染退城、清水润城、城市增绿，加快建设生态唐山实现绿色发展

一是以污染退城倒逼产业转型，根治环境顽疾。主动超前化解出清过剩产能，推动总量去产能向结构去产能转变。强化排查监管，严控新增违规钢铁产能，严防封停装备复产，严禁“地条钢”死灰复燃。推动主城区周边工业企业沿海布局、临港临铁发展。切实担当起京津冀生态环境联建联防联治的唐山责任，全面执行严于国标省标的“唐山限值”。持续实施错峰生产运输，分类建立差异化管控清单。年内PM2.5平均浓度下降5%，单位工业增加值能耗下降4%以上。二是以清水润城修复河流湖库，提升生态质量。统筹推进“治用保管”，实施6大类47个全域治水项目，年内国省考核断面全部达标，全域消除劣五类和黑臭水体。加快河道治理、河湖连通。强化水源涵养及供水保障，加大地下水超采治理力度，启动市中心区污水处理厂迁建提标，22个县级污水处理厂出水水质全部达到类四类标准。严格落实河湖长制，强力推进河道矿山尾矿综合治理。深度开展监测分析、溯源倒查、统筹施治等各项工作，重塑岸绿滩美自然景观。三是以城市增绿筑牢生态屏障，拓展环境容量。以“高大厚密彩”为标准，大力推进“一街一景”“见缝插绿”“增绿添彩”，完成重点道路沿线76个单位庭院和46个居民小区增绿建设。高标准提升环城高速两侧各500米绿化带。狠抓农用地、建设用地污染风险管控，推动城乡垃圾分类和固体废物资源化利用，依法严厉打击非法处置危险废物、排放有毒有害物质等行为。深入开展国土绿化和矿山迹地整治，成功创建国家森林城市。

9.深入实施乡村振兴战略，加快农业农村现代化

一是坚决打赢精准脱贫攻坚战。坚持现有目标标准不动摇，在全省率先实现高质量整体脱贫。深入推进产业、就业、教育、健康、社会保障等扶贫，组织开展“百企帮千户”活动。建立完善精准防贫长效机制，强化脱贫户后续扶持，巩固和扩大脱贫成果。二是壮大特色农业新优势。深化农业供给侧结构性改革，力争创建省级国际标准农产品生产示范区15个，擦亮“安全食品唐山造”名片，支持引导社会资本投资休闲农业、乡村旅游、农村电商等新产业新业态。三是改善农村人居环境。强力实施农村人居环境整治三年行动，突出抓好污水垃圾处理、“厕所革命”“四好农村路”建设。深入开展农村环境“百村示范、千村提升”行动，推动农村移风易俗，营造充满活力、崇德向善的淳美乡风。

10.用心用情增进民生福祉，让群众有更多、更直接、更实在的获得感幸福感安全感

一是办好人民满意教育。聚焦解决学前教育“入公难、入民贵”问题，聚焦解决义务教育“乡村弱、城镇挤”问题，年内全部消除“超大班额”。聚焦高中教育提质量、创品牌，持续开展“三名”工程。聚焦产教城深度融合，支持华北理工大学对标“双一流”建设，唐山工业职业技术学院、唐山职业技术学院创建国家优质高职院校。提升唐山师范学院、唐山学院办学水平。二是满足人民群众多层次健康需求。优化医疗资源布局和服务能力，鼓励引导社会资本依托医疗资源，加快发展养老、康复、保健、育婴等产业。提高城乡居民医疗保险惠民待遇，降低群众就医成本，基本医疗保险三级医院支付比例由55%提高至60%，转外住院和异地急诊住院支付比例提高5个百分点、起付标准由2500元降低至1200元。三是全面提升保障水平。强化创业带动就业，重点培育30家创业企业，确保全市新增城镇就业12万人以上。坚持“房住不炒”定位，新开工保障性安居工程2.5万套。城乡居民最低生活保障标准分别提高至每人每月666元、每人每年4824元。巩固国家食品安全示范城市成果，市级“放心肉菜示范超市”达到50家以上，打造百姓放心的“菜篮子”。大力实施文体惠民工程，创建国家公共文化服务体系示范区。四是深化平安唐山建设。牢牢把握风险防控主动权，排查整治经济、金融、政府和国有企业债务、房地产等领域风险隐患。深入开展危化品、道路交通、建筑施工、消防、人员密集场所等行业领域专项整治。开展药品保健品非法营销专项整治。深入落实习近平总书记“两个坚持、三个转变”防灾减灾救灾重要指示精神，加快中国·唐山防震减灾示范中心建设，打造国内一流、国际知名的防震减灾示范市。扎实有序推进信访工作，妥善解决群众合理诉求。纵深推进扫黑除恶专项斗争，完善共建共治共享社会治理格局，升级“智慧公安”建设工程，争创全国社会治安综合治理优秀市。加强国防动员和后备力量建设，维护军人军属合法权益。围绕创建全国双拥模范城市，做好退役军人移交安置、抚恤优待、教育培训等工作，落实各项政策，提高管理服务水平，在全社会营造尊崇尊重军人的浓厚氛围。积极发展老龄、残疾人、妇女儿童、关心下一代事业，统筹做好民族宗教、广播电视、新闻出版、外事侨务、人民防空、老区建设、邮政快递、气象、档案等各项工作。
